# Microstructure and Defect-Based Fatigue Mechanism Evaluation of Brazed Coaxial Ti/Al_2_O_3_ Joints for Enhanced Endoprosthesis Design

**DOI:** 10.3390/ma14247895

**Published:** 2021-12-20

**Authors:** Johannes L. Otto, Ivan Fedotov, Milena Penyaz, Thorge Schaum, Anke Kalenborn, Boris Kalin, Oleg Sevryukov, Frank Walther

**Affiliations:** 1 Chair of Materials Test Engineering (WPT), TU Dortmund University, 44227 Dortmund, Germany; thorge.schaum@tu-dortmund.de (T.S.); anke.kalenborn@tu-dortmund.de (A.K.); frank.walther@tu-dortmund.de (F.W.); 2 Department of Materials Science, National Research Nuclear University MEPhI (Moscow Engineering Physics Institute), 115409 Moscow, Russia; IVFedotov@mephi.ru (I.F.); MAPenyaz@mephi.ru (M.P.); ONSevryukov@mephi.ru (O.S.)

**Keywords:** brazing, titanium, ceramic, alumina, implants, fatigue, strength, computed tomography, pores, fractography

## Abstract

Alumina-based ceramic hip endoprosthesis heads have excellent tribological properties, such as low wear rates. However, stress peaks can occur at the point of contact with the prosthesis stem, increasing the probability of fracture. This risk should be minimized, especially for younger and active patients. Metal elevations at the stem taper after revision surgery without removal of a well-fixed stem are also known to increase the risk of fracture. A solution that also eliminates the need for an adapter sleeve could be a fixed titanium insert in the ceramic ball head, which would be suitable as a damping element to reduce the occurrence of stress peaks. A viable method for producing such a permanent titanium–ceramic joint is brazing. Therefore, a brazing method was developed for coaxial samples, and two modifications were made to the ceramic surface to braze a joint that could withstand high cyclic loading. This cyclic loading was applied in multiple amplitude tests in a self-developed test setup, followed by fractographic studies. Computed tomography and microstructural analyses—such as energy dispersive X-ray spectroscopy—were also used to characterize the process–structure–property relationships. It was found that the cyclic loading capacity can be significantly increased by modification of the surface structure of the ceramic.

## 1. Introduction

**Figure 1:**
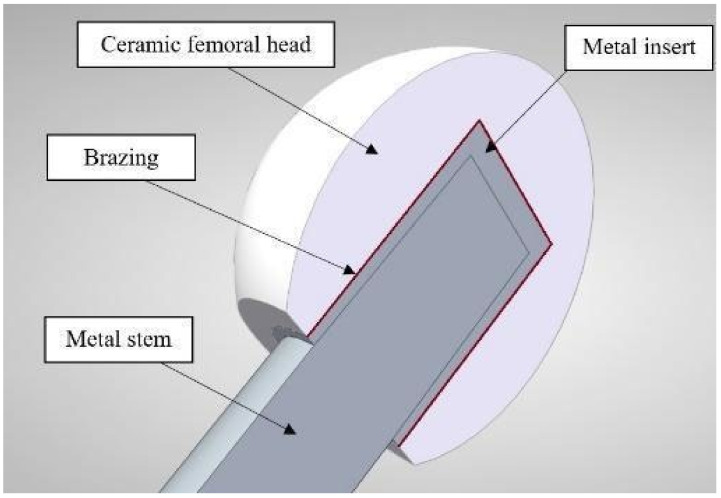
Design of a hip joint endoprosthesis femoral head with an integrated metal insert.

Advances in medical implants over the past few decades have led to their widespread use in treating various types of pathologies and injuries, but they still hold potential for further development. The replacement of the hip joint with an endoprosthesis is one of the most frequently performed surgeries in traumatology, and can make a substantial contribution to increasing a patient’s quality of life. Thus, a key aspect of sustainable patient treatment is the durability and reliability of the endoprosthesis. Currently, there are four major types of friction pairs applied in hip joint endoprosthesis: metal-on-metal (MoM), metal-on-polyethylene (MoP), ceramic-on-polyethylene (CoP), and ceramic-on-ceramic (CoC) [1]. The use of a MoM pair head and cup, made from metal alloys, can lead to tribocorrosion and the release of microscale metal debris and ions into the surrounding tissues [2]. Implants made of polyethylene inserts and a metal femoral head have issues with wear resistance, making them strongly dependent on the quality of the raw materials and the sterilization method for the polyethylene [3]. In comparison, hip joint endoprostheses with CoC or CoP friction pairs have numerous advantages in terms of biological inertness, a very low wear rate, and the possibility of polishing the ceramic to a very fine roughness for a uniform distribution of synovial fluid [4]. Nevertheless, there is always a risk of a femoral head breaking due to defects in the ceramic and/or excessive shock loading, leading to a heavy revision hip replacement [5]. To avoid these problems, a design of the endoprosthetic femoral head with an integrated metal insert is proposed (Figure 1).

The main objective of the metal insert is to prevent the formation of high stress peaks in the ceramic element, thereby increasing its reliability. As the base material for femoral head manufacturing, near-pure aluminum oxide (Al_2_O_3_/alumina) was used in recent decades for endoprosthetic ceramics such as Biolox^®^ or Biolox^®^ forte, but today variations of zirconia-toughened alumina (ZTA) are mostly used for endoprosthetic ceramics such as Biolox^®^ delta, due to their increased mechanical properties [5,6]. However, pure alumina was chosen for this study, as it is suitable for demonstration purposes, and no specific ceramic trademark was used. Pure titanium (cp-titanium) and its alloys, such as Ti-6Al-4V, are well-known biocompatible materials for permanent implants [7]. The joining of alumina with metal for biomedical applications can be carried out in two different ways: brazing, or adhesive bonding. However, because of the degradation of adhesives over time, brazing is expected to show a higher potential for long-term applications. The choice of filler metal for brazing is mainly dependent on the chemical and physical properties of the base materials, and in the case of biomedical implants it should not contain harmful elements. The release of ions can cause allergic reactions or other serious health consequences with several elements. Therefore, the design of a biocompatible filler material for the brazing of titanium and alumina ceramics is a key challenge for this development.

Brazing of titanium and alumina can be maintained with different filler metals and brazing modes. In [8], Al_2_O_3_/Ti bonding was carried out with pure gold in a vacuum at a 1100 °C brazing temperature. This method achieves a strong bonding, but causes high costs in case of large brazing areas. Another method entails brazing with Zn–5Al [9] filler metal and preliminary metallization with the same alloys in a bath under ultrasonic waves; the main disadvantage of this method is the high content of aluminum, which has been found to be a harmful element for bones and neurons [10]. 

The rapidly quenched filler metal based on a Zr–Ti system appears to be a good way to braze ceramics with metal alloys [11]. These alloys always contain elements that cause lower melting temperatures of the filler metal (depressants), e.g., Ni, Cu, and Be. Nickel can be very allergenic [12], and the excess of copper and the presence of beryllium in the human body can lead to various diseases [13]. The only way to achieve a suitable melting temperature for filler metals is to use cobalt as a depressant element, since cobalt is widely used for femoral head manufacturing in MoM friction pairs. 

The aim of this work was the brazing of highly fatigue-resistant coaxial alumina/titanium joints with a Zr–35Ti–26Co filler metal and the investigation of macro- and microstructure, as well as the evaluation of fatigue properties to identify relevant process–structure–property relationships for further development steps. For this purpose, surface modifications of the ceramic need to be done, which will be the subject of further research.

## 2. Materials and Methods

### 2.1. Brazing

Commercial alumina ceramic (ELC-99.7, Elemet, Moscow Oblast, Russia) and titanium (Russian grade VT1-0, analogous to Ti Grade 2, Auremo OOO, Saint Petersburg, Russia) were used as base materials for brazing. Their composition according to the manufacturers’ data is given in Table 1. In addition, some selected properties of the materials are given in Table 2, which should support the understanding of their production and application.

**Figure 2:**
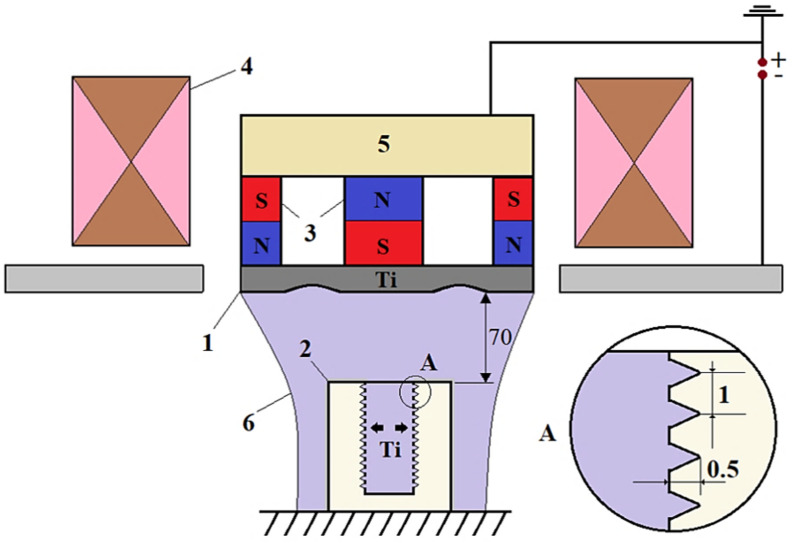
Scheme of magnetron sputtering: 1—sputtering cathode; 2—ceramic sample; 3—permanent magnets; 4—electromagnet; 5—magnetic circuit; 6—sputtered titanium.

To improve the fatigue resistance of the joint, the contact area of the brazed joint should be extended and a form-fit between the components created. For this purpose, the inner surface of the ceramic cylinder was milled with a diamond tool. The profile was produced in the form of triangular/elliptical notches with a depth of 0.5 mm and a spacing of 1 mm. After the milling treatment and prior to vacuum brazing, alumina cylinders were annealed in an air muffle furnace at 1000 °C for 3 h. Additionally, a titanium coating was sputtered onto the inner (brazing) surface of the ceramic cylinders using the magnetron deposition method, as shown in Figure 2, in order to increase the wettability of the ceramic. The working parameters of the deposition were chosen so as to obtain a coating with a thickness of ~1 μm. To validate the improved fatigue properties, this study compares the properties of coaxial brazed alumina/titanium joints with and without these modifications of the ceramic.

As already mentioned, a Zr–35Ti–26Co (wt.%, or Ti_45_Zr_27_Co_28_, respectively) alloy was selected as the filler metal for the alumina/titanium brazing. The composition of the selected alloy was in the range of the probable minimum melting point in the Ti–Zr–Co system [16], which is favorable for brazing a ceramic/metal joint with low, thermally induced residual stresses. Initially, the filler metal was obtained in the form of ingots via argon arc melting using commercially pure titanium, zirconium, and cobalt. The tape of the filler metal was made using a melt-spinning technique on the Crystal-702 installation. The final step of the filler metal preparation was the embrittlement of the tape by annealing in a vacuum furnace (XRetort, Xerion Berlin Laboratories^®^ GmbH, Berlin, Germany) at 5·10^−3^ Pa for 2 h at 500 °C, followed by further grinding in a planetary mill in order to obtain a powder with particle size of 50–350 μm.

**Figure 3:**
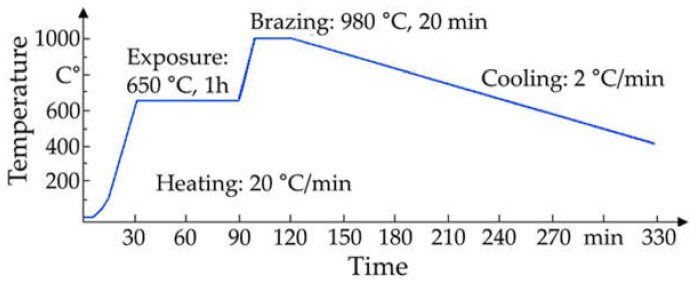
Time–temperature brazing mode.

**Figure 4:**
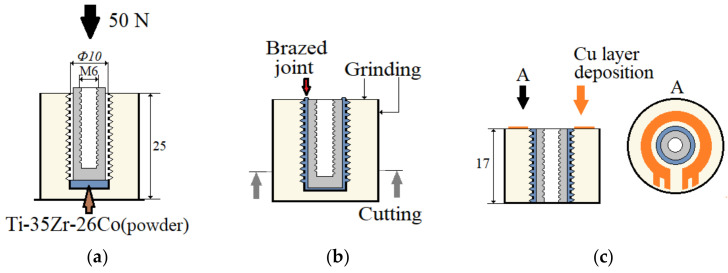
Alumina/titanium brazing and processing cycle of a milled and sputtered sample: (**a**) brazing; (**b**) post-processing; (**c**) spraying contacts.

The brazing of the coaxial alumina/titanium joints was carried out in a vacuum furnace (Xerion Xvac 1600, Xerion Berlin Laboratories^®^ GmbH, Berlin, Germany) at less than 5·10^−3^ Pa. The brazing mode graph is shown in Figure 3, and the sample preparation in Figure 4. The Zr–35Ti–26Co filler metal was placed on the bottom of the ceramic sample with 0.8 g. The titanium insert with an M6 blind thread hole was placed on top of the filler metal and inside of the ceramic cylinder. During the brazing process, a static load of 50 N was applied to the titanium insert to extrude the molten filler metal into the coaxial gap.

After brazing, the samples of the alumina/titanium joint were cut from the bottom side, and the top and side surfaces were ground. This step was necessary since, in this study, only the properties of the coaxial brazed joint were investigated, as described in Section 2.3. An additional deposition of a copper layer for the contact of electrical measurement techniques, applied during fatigue testing, was carried out via magnetron sputtering, using the same method as for the titanium deposition, for a few selected samples. The operating parameters of the deposition were chosen so as to obtain a copper coating with a thickness of ~10 μm.

### 2.2. Macro- and Microstructure Investigations

For microstructure studies, samples were first cut with a diamond precision disc, hot-mounted, ground, and polished. The surface was finished with an oxide polishing suspension (OPS). For scanning electron microscopy (SEM), a backscattered electron (BSE) detector was used for high material contrast. Energy-dispersive X-ray spectroscopy (EDX) was performed in the form of mapping with an FIB-SEM (Crossbeam 550, Zeiss, Oberkochen, Germany) in order to obtain information about elemental distribution. 

**Figure 5:**
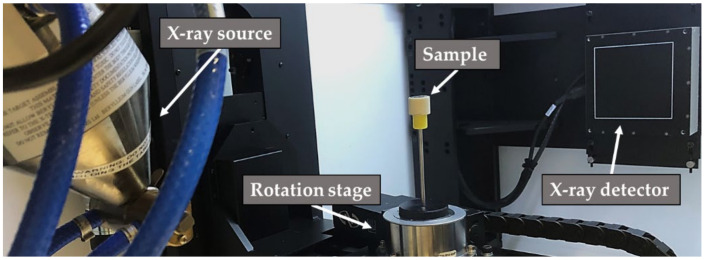
Setup of CT scanning with sample.

Computed tomography (CT) examinations were conducted using an X-ray computed tomography scanner (XT H 160, Nikon Metrology, Leuven, Belgium). The system enables an investigation with a maximum voltage of 160 kV and a maximum power of 60 W, that allows voxel sizes down to 3 µm due to the 1008^2^ pixel detector. The setup of the scanning is presented in Figure 5. Volume reconstruction and defect analysis of the CT scans were performed using a visualization and analysis software (VGStudioMax 2.2.4, Volume Graphics GmbH, Heidelberg, Germany). The aim of our analysis was to detect internal defects inside the brazing material, as well as inside the ceramic. To compare internal defects and defect distribution with mechanical loads, all samples were CT scanned before and after mechanical testing. The scan parameters were adjusted to focus on the ceramic compared to a focused observation of the brazing material. The parameters shown in Table 3 achieved the best results in terms of expected image quality.

### 2.3. Mechanical Testing 

**Figure 6:**
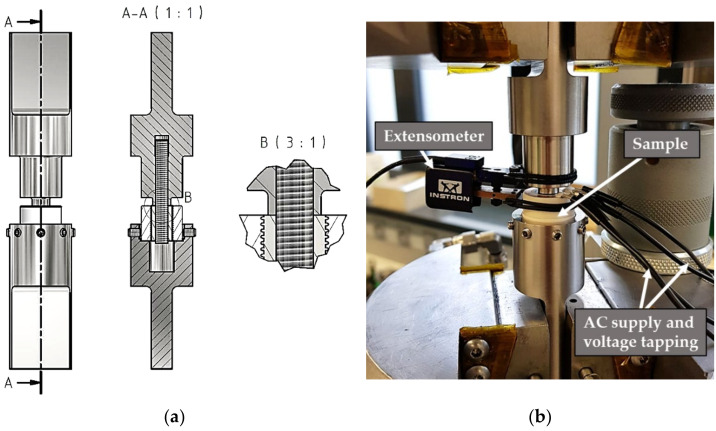
(**a**) Technical drawing of the clamping device for samples with cross-sectional view (A-A) and detailed view (B), and (**b**) test setup for fatigue tests using the servo-hydraulic testing system.

A servo-hydraulic testing system (8801, Instron, Norwood, MA, USA) with a maximum dynamic load capacity of ±100 kN was used to perform the fatigue testing. A clamping device was developed—which can be seen in Figure 6a—to fix the samples for carrying out the tests in the compression range, which means that no tensile force was applied. By using a threaded rod inside them, the samples were centered. The main part of the load was applied directly to the titanium insert from above, via the upper part of the clamping device, like a stamp, ensuring that the brazed seam was tested instead of the thread (this would happen if only a threaded rod was used to transmit the load). The lower part of the clamping device was centered by using grub screws, with a narrow strip of adhesive tape applied to the side in order to protect against unwanted fixation-related stress peaks. Since no tensile force was applied, no other fixation was needed. The ceramic cylinder cannot change its position because it stands on the lower part of the fixture, but the lower part has a hole that is slightly larger than the titanium insert; this allows the titanium insert to be pushed down if the brazed seam or the ceramic fails, while the ceramic does not move. A maximum push-in of the titanium insert by 0.8 mm was set as the failure criterion on the fatigue test system. The whole setup, including the used measurement techniques, is presented in Figure 6b. Most important is the use of an extensometer with a gauge length of 10 mm; this allows measurement of the displacement of the titanium insert against the surrounding ceramic in form of the total mean strain, and gives further information about material reactions—such as damage progression of the brazed seam—by the total strain amplitude. Additionally, an alternating current potential drop (ACPD) approach was applied for crack monitoring on the upper side of the ceramic on selected samples, which were prepared as shown in Figure 4c. The aim of this was to find out whether the brazed seam or the ceramic failed first. A constant current was applied and the voltage was measured. As a result of a damaged sputtered copper ring and the associated increase in resistance, the voltage must increase at a constant current.

Time-efficient and sample-saving multiple amplitude tests (MATs) were used as a test procedure, allowing for the evaluation of the fatigue behavior and comparison of different brazing processes [17,18]. The validation of test results by S–N curves is planned for further studies. All tests were performed with a frequency f = 10 Hz and a stress ratio R = 10 (negative loads/compression only). MATs were performed with a stepwise increase of ΔF_max_ = 0.25 kN or ΔF_max_ = 0.5 kN after each ΔN = 10^4^ cycles, until failure of the samples.

## 3. Results

### 3.1. Macrostructure

**Figure 7:**
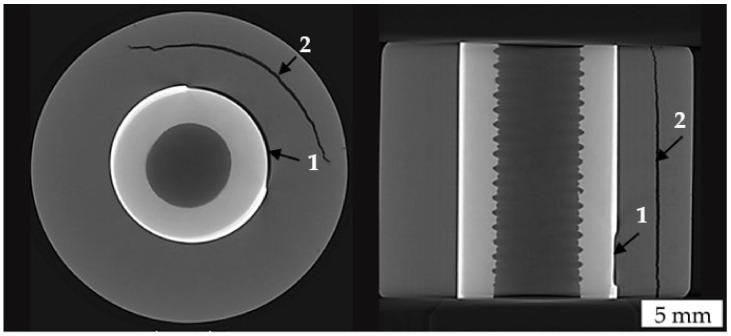
Horizontal and vertical cross-sectional views of a non-sputtered and unmilled specimen showing a non-brazed area (1) and a large crack (2) after brazing.

Most samples that were not milled and not sputtered showed clearly visible radial cracks in the ceramic cylinder after brazing. Since the thermal expansion coefficients of titanium and alumina are not equal, it is a known challenge to avoid cracks, because of the strong thermally induced residual stresses that arise from the cooling process and the dissimilar shrinking behavior [19,20]. Tensile forces are especially critical for ceramics [20]. Therefore, a slow cooling rate was used, and different component geometries were tested in previous development steps. However, according to the parameters described in Section 2.2, it was possible to examine the ceramic in the CT in order to investigate this phenomenon. Figure 7 shows the horizontal and vertical cross-sectional views of such a non-sputtered and unmilled specimen with a radial crack extending over almost the entire height of the sample. The ceramic appears darker due to its lower density ρAl_2_O_3_ = 3.95 g/cm^3^, while the brazing powder is significantly lighter compared to the titanium, with ρ_Ti_ = 4.50 g/cm^3^, due to the higher density of zirconium ρ_Zr_ = 6.51 g/cm^3^ and cobalt ρ_Co_ = 8.90 g/cm^3^ [14]. All scanned samples with cracks in the ceramic showed non-brazed areas (marked with 1) that were often located near the visible cracks (marked with 2). Therefore, it can be assumed that these brazing defects lead to an inhomogeneity of the otherwise uniform axisymmetric residual stress distribution during the cooling, and that certain parts of the ceramic cylinder are subjected to critical tensile stresses leading to the observed cracks. In the samples that were milled and sputtered, such large non-brazed areas and clearly visible cracks in the ceramic could not be found. This could be related to the improved wettability due to the sputtered titanium layer, whereby the profiling could also have influenced the flow behavior of the liquid brazing alloy during brazing [21]. Uneven areas with reduced gap sizes in the ceramic could be better wetted if additional flow paths are created by the profiling. 

**Figure 8:**
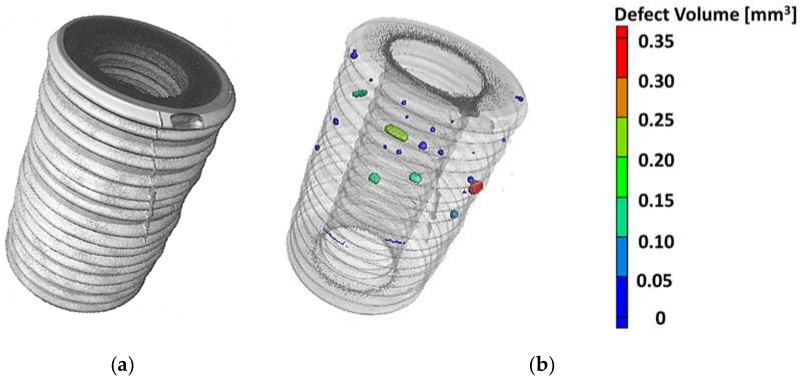
(**a**) 3D representation of the titanium insert, and (**b**) transparent representation with pore analysis, of a milled and sputtered sample.

Figure 8a shows a CT scan focused on the titanium insert and the brazed seam by using a higher beam intensity and current of a milled and sputtered sample. The remaining ceramic pixels were masked out during image processing, making the bonding surface of the brazed seam visible. Some defects are already visible here; for example, a fine vertical crack seems to have formed inside the ceramic, which has filled with the brazing alloy. Moreover, a larger pore on the upper side of the sample is noticeable here. However, analysis of the enclosed pores within the brazed seam provides further important information, which can be seen in Figure 8b, as most of the pores are found here. The detected pore sizes vary from 1.3 × 10^−4^ to 3.4 × 10^−1^ mm^3^. An increased accumulation of pores in the upper part of the sample can be clearly seen, which must be explained by the brazing process, since gas inclusions must rise in order to leak out and escape into the vacuum. If this process is not completed sufficiently quickly, the pores can become fixed in the brazed seam as it begins to solidify.

### 3.2. Microstructure

**Figure 9:**
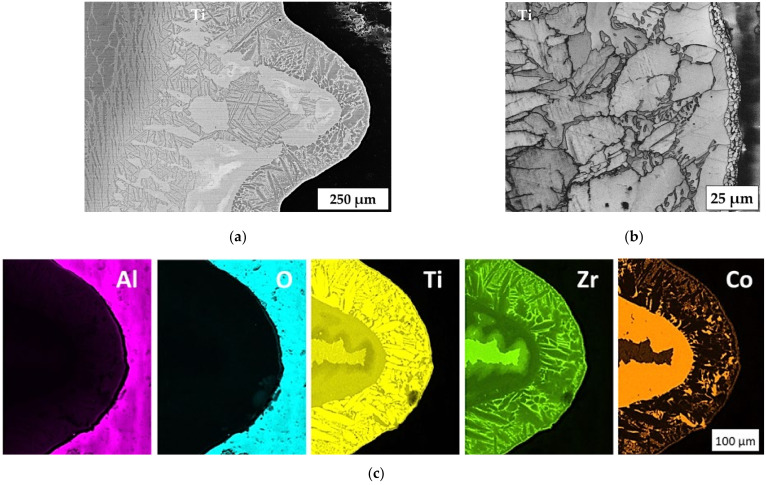
(**a**) SEM-BSE image at magnification of 100×, (**b**) EBSD band contrast image at magnification of 500×, and (**c**) EDX mapping at magnification of 250× with 15 kV of the brazed seam in a notch located near the bottom side.

Figure 9a shows an SEM-BSE image of a milled and sputtered sample at a magnification of 100× of a notch at the lower part of the sample close to the bottom. Figure 9b represents an EBSD band contrast image at magnification of 500× focused on the top of such a notch in the same area. Figure 9c presents the elemental distribution by EDX mapping at a magnification of 250×, detected with 15 kV. The brazed joint can be divided into different areas. The first zone, close to the ceramic, consists of a layer of 2–3 very small grains with a grain diameter of ~0.5 to 2 µm, which is caused by sputtering. According to the EDX images, this zone is enriched with zirconium and cobalt, as these elements diffused into the sputtered layer during brazing. 

The interaction zone of the filler metal with titanium was estimated to be ~300 µm, whereas the initial gap between the ceramic element and the titanium pin was no more than 100 µm. This indicates the interaction of the filler metals with titanium, as a result of which crystallization begins (Figure 9).

It can be seen from Figure 9b that equiaxial crystals are formed on the alumina/brazed joint interface, and then columnar crystals are crystallized in the titanium direction. The distribution of the elements of the brazed seam shows that there are three further zones, each enriched with their own elements: titanium, zirconium, and cobalt (Figure 9c). Columnar crystals mainly contain titanium, whereas the space between the crystals contains zirconium. Inclusions of cobalt-enriched phases were also found in the brazed joint, which probably correspond to the formation of intermetallic compounds.

**Figure 10:**
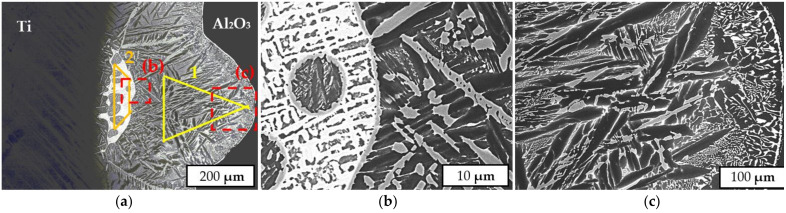
SEM-BSE images of the brazed seam in a notch located in the center of the sample, showing the general microstructure (**a**), a dendrite crystal zone (**b**) and a columnar crystal zone (**c**).

Another structure of the brazed joint was observed in the central zone of the sample, where the cooling rate was lower due to the lack of contact with the substrate on which the sample stood. In the zone close to the ceramic, shown in Figure 10c, the columnar structure of crystallites prevails, whereas structures with dendritic crystals are observed in the central zone of the brazed seam closer to the titanium. Table 4 indicates the results of an EDX zone analysis of the brazed joint in the area of the formation of columnar and dendritic crystals.

The average cobalt content in the brazed joint zone corresponding to the formation of columnar crystals was 7.6 at.%, while the content in the dendritic crystal zone was 14.7 at.%. Compared to the filler metal, the cobalt content in the seam was lower, which may indicate its diffusion into the titanium at the brazing temperature. The presence of aluminum in the brazed joint is related to the interaction of the filler with aluminum oxide, during which it dissolves.

### 3.3. Fatigue Behavior 

**Figure 11:**
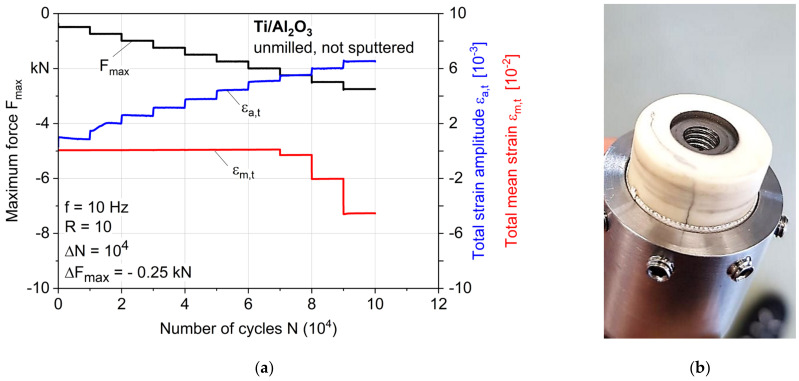
(**a**) Results of a multiple amplitude test (MAT), and (**b**) failed sample without milled and/or sputtered ceramic.

Figure 11a shows the MAT results of a sample whose ceramic was neither milled nor sputtered with a titanium layer before brazing. The maximum pressure force was increased in small steps with ΔF_max_ = −0.25 kN, since a weak bonding was expected. The total mean strain stayed nearly constant until −2.25 kN was reached. Within the next three steps, the total mean strain increased rapidly until the sample failed at −2.75 kN after 1·10^5^ cycles. The total strain amplitude shows an almost inversely linear dependent course to the maximum force, indicating no specific fatigue behavior. Irregularities during the first two steps of the test could be explained by low forces and slight unevenness of the titanium, whereby the upper part of fixture could not yet use the full contact area. Figure 11b shows the titanium insert that was just pushed through the ceramic without damaging it; the visible cracks appeared already after brazing (see Figure 7, same sample), so the brazed seam can be clearly identified as being the weakest point of the joint.

**Figure 12:**
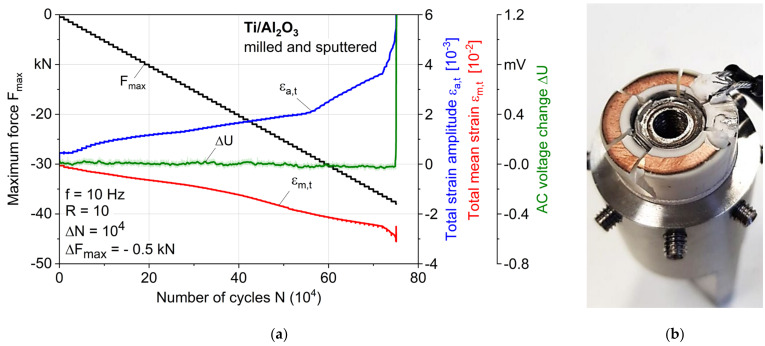
(**a**) Results of a multiple amplitude test (MAT), and (**b**) failed sample with milled and sputtered ceramic.

Figure 12a shows the MAT results of a sample whose ceramic was milled and sputtered as described in Section 2.1 before brazing. The maximum pressure force was increased in greater steps with ΔF_max_ = −0.5 kN, since a stronger bonding was expected. Considering the obtained curve of F_max_, it is obvious that this step size was still small, because the maximum force up to the failure of the sample with F_max_ = −37.5 kN was increased by a factor of more than 13 compared to the previous shown tests (note the change in the maximum force scale by a factor of five). The total mean strain shows a course that changes mainly due to the increase in force, and only shows a decrease a few steps before failure, which indicates the beginning of the growth of fatigue cracks. This is also reflected by the significant increase in strain amplitude at the same number of cycles at around F_max_ = −36 kN. The nonlinear progression within each step shows a cycle-dependent damage behavior of the joint from this point on. An earlier increase in the total strain amplitude could be related to the initial debonding effects of the brazed seam without leading to a displacement of the titanium insert, or to microstructural effects such as the softening of the titanium. The AC voltage change showed a strong increase only in the last 270 cycles; beforehand, there was only a noise due to the electrical setup, mainly because of the low current, which is difficult to regulate and was needed for the thin copper layer. This means that a vertical crack in the ceramic reached the sputtered ring of copper shortly before the final fracture, and then propagated fast and brittle within 270 cycles. Hence, none of the other signals of the extensometer that were measured previously could be related to vertical cracks in the ceramic; therefore, changes in the strain must be related to material reactions of the brazed seam, internal damage accumulation of the base materials, or any other kind of cracks in the ceramic (which were not visible), and do not lead to failure of the sample. Figure 12b presents the failed sample, which clearly shows a final fracture of the ceramic; therefore, the brazed seam seems not to be the weak point of this joint anymore. 

### 3.4. Fractography

**Figure 13:**
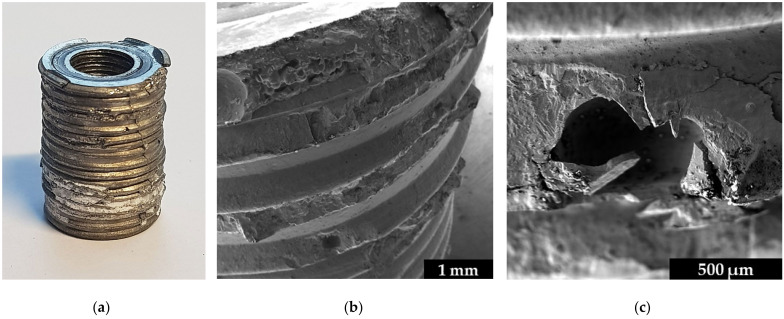
Fractographic images of the whole titanium insert, including (**a**) ceramic fragments, (**b**) the failed brazed seam in the upper part, and (**c**) a broken pore.

The ceramic fragments of the failed MAT sample shown in Figure 12b were removed, and the damaged titanium parts (titanium insert and brazed seam) are presented in Figure 13a. It can be seen that in the lower area with few pores (compare Figure 8), ceramic fragments clearly adhere, while in the upper area of the sample with more pores, more titanium has failed. In addition, some fractures of the titanium can be seen on top of the sample at the transition from titanium to ceramic where the punch of the clamping device introduced the force, but this test-setup-dependent effect is limited to a small area of the brazed seam. Thus, it can be assumed that the presence of the pores is an important factor that shifts the failure mechanisms to the point where the brazed seam has a higher or lower fatigue strength than the ceramic. This assumption is supported by further fractographic studies at higher magnification in the SEM shown in Figure 13b,c. The pores seem to initiate local cracks, which can lead to the fracture of the entire negative imprint of the notch through the seam. 

## 4. Discussion 

### 4.1. Crystallization Mechanism

The examinations in Figure 9 and Figure 10 show that a heterogeneous microstructure was formed in the brazed joint. The results of EDX analysis and comparison with the Ti–Co phase diagram show that the average cobalt content in the zone of columnar crystals corresponds to the eutectoid point. This means that this zone corresponds to β-titanium at the brazing temperature. This type of brazed joint microstructure (Widmanstaetten) is widespread in brazing with titanium filler metals at temperatures above the α-Ti(Zr) → β-Ti(Zr) transformation [22]. 

The selected filler metal composition corresponds to the melting temperature (liquidus) not being higher than 900 °C [23]. Therefore, it was assumed that the filler metal was completely liquid at the brazing temperature, and flowed into the gap between the alumina and titanium until the dissolution and further diffusion of titanium in the brazed seam finally led to the formation of the first crystals of β-(Ti,Zr). 

During the cooling phase, a crystallization front was formed on the side of the ceramic due to heat transfer to the external environment. The microstructure of the columnar crystals shows that their growth occurred on the titanium side, pushing the excess cobalt in the liquid filler metal to the concentration of the possible eutectic reaction:

L → (Ti,Zr)_2_Co + β-(Ti,Zr)

The formation of a zone with dendritic crystals in the titanium boundary zone may be related to the described process, since the instability of one of two eutectic phases can lead to the formation of a microstructure composed of dendrites and eutectics [24]. Further cooling results in the eutectoid reaction in the β-(Ti,Zr) phase, which leads to the formation of lamellar (Widmanstaetten) structures in the entire area of the brazed joint:

β-(Ti,Zr) → (Ti,Zr)_2_Co + α-(Ti,Zr)

However, in order to determine the exact phase composition of the brazed joint, and to confirm the assumptions made about the mechanism of crystallization of the brazed joint, more detailed EDX, EBSD, and XRD studies are needed, which will be the subject of future works.

### 4.2. Fatigue Behavior and Mechanisms 

Simulated loading cycles for hip implants with a body weight of 100 kg show a maximum resulting force of −2.880 kN for the activity *walking* and −4.839 kN for *jogging* [25]. According to ISO 7206-6 [26], for the fatigue testing of a hip implant’s stem neck, a maximum force of −5.340 kN should be applied sinusoidally until 5 × 10^6^ cycles. Compared to the maximum force achieved in MATs of −37.5 kN until failure, the presented coaxial Ti/AL_2_O_3_ joint is expected to show sufficiently high fatigue strength. Considering the real endoprosthesis geometry, a part of the force is also applied to the bottom of the titanium insert (which was removed in this study so as to investigate only the brazed seam), thus further reducing the locally acting forces. Nevertheless, further fatigue tests until 1·10^7^ cycles with lower forces are planned to complete the fatigue characterization. 

All mechanical tests (Section 3.2) were repeated for verification, and an even higher difference in fatigue strength for both tested sample types—of more than a factor of 16 (2.5 to 40.5 kN)—could be achieved, underlining the need for surface modifications of the ceramic for a high fatigue strength. However, due to the complex brazing process, varying sample qualities with regard to pores or small ceramic cracks made a direct comparison within the samples of the same parameters difficult. This shows that further process optimization is needed for a high, reliable joint quality, and the quality and condition of the raw materials should be monitored, since the joint quality largely depends on them. At the same time, it must be investigated and defined what the critical defect sizes are regarding crack initiation for application-relevant loads. Completely defect-free joints will probably not be producible, highlighting the need for a detailed defect and damage tolerance evaluation.

### 4.3. Futher Investigations 

In addition to the characterization of the brazed seam, investigations on the final cubic ceramic head with the titanium insert are required in the next steps in order to verify, for example, the damping properties of the titanium insert, as already shown by simulation [27]. For this purpose, digital image correlation (DIC) or local strain measurements with strain gauges should be used, as already shown in combination for brazed steel joints in [28]. For further qualification of the brazed joints for use in the human body, biocompatibility tests are essential. In particular, the ion release of the individual elements in liquid media should be investigated [29,30], including the release under cyclic loading. 

In addition, the transferability of the results to other ceramic materials should be examined, for which ZTA should be used, which is widely used for endoprosthetic applications. Differences in CTE may require adjustments to the brazing process but, on the other hand, the better fracture strength may also help to avoid thermally induced stress cracking. In any case, studies of residual stresses need to be performed using an X-ray diffractometer for a better understanding regarding the influence of process parameters.

## 5. Conclusions

The present study was able to demonstrate that brazing of coaxial Ti/AL_2_O_3_ joints with high fatigue strength using a Ti–Zr–Co brazing powder is possible with suitable ceramic surface preparations. It was shown to be important that a form-fit joint is created, allowing for a high force transmission of shear forces from the titanium into the ceramic, for which circumferential elliptical notches at the ceramic surface have proven suitable. The notches in combination with a sputtered titanium coating apparently improved the flow behavior of the melted filler metal, as shown by the comparison of CT scans of samples with and without surface treatments. The latter samples often had unbrazed areas with significant cracks, which could probably attributed to an uneven stress distribution. Investigations regarding the effects of pores identified in the upper sample region on the fatigue mechanisms showed that the reduction in the number and size of pores is necessary in order to further increase the fatigue performance of the joint. Relevant process–structure–property relationships could be identified, providing an important contribution to further developments towards an enhanced endoprosthesis design with brazed components. 

## Figures and Tables

**Table 1:** Chemical composition of the used components.

Components	Al_2_O_3_	Ti	Zr	Co	Other/Impurity
Filler metal powder	-	35.0	39.0	26.0	-
Alumina cylinder	99.7	-	-	-	0.3
Titanium insert	-	99.2–99.7	-	-	0.3–0.8

All values in wt.%.

**Table 2:** Selected properties of the materials, according to the manufacturers’ data and [14,15].

Material	CTE(0–100 °C) (µm/m/°C)	Young’s Modulus (GPa)	Tensile Strength (MPa)	Fracture Toughness (MPa m^1/2^)	Hardness HV 10
Alumina (~99.7%)	7.5–8.5	380	300–470	4.0	2200
Titanium (VT1-0)	8.5–8.6	112	355–540	60.0	140–170

**Table 3:** CT scan parameter.

Focussed Object	Beam Intensity	Beam Current	Exposure Time
Ceramic cylinder	106 kV	70 µA	500 ms
Titanium insert, brazed seam	125 kV	74 µA	500 ms

**Table 4:** Results of EDX area analysis from Figure 10.

Zone/Element (at. %)	Al	Ti	Co	Zr
1	1.4	80.4	7.6	10.6
2	0.7	68.2	16.4	14.7

## Data Availability

The data presented in this study are available upon request from the corresponding author. The data are not publicly available due to privacy.
